# Supplementary material for: Visible-light-switched electron transfer over single porphyrin-metal atom center for highly selective electroreduction of carbon dioxide
Source: Nat Commun. 2019 Aug 26;10:3844. doi: 10.1038/s41467-019-11817-2 (PMC6710284; doi:10.1038/s41467-019-11817-2)
Supplement: Supplementary file 1 — Supplementary Information [file 41467_2019_11817_MOESM1_ESM.pdf]

Supplementary Information

Visible-light-switched electron transfer over single porphyrin-metal atom center for the highly selective electroreduction of CO_2

Deren Yang[1], Hongde Yu[1], Ting He[1], Shouwei Zuo[2], Xiaozhi Liu[3], Haozhou Yang[1], Bing Ni[1], Haoyi Li[1], Lin Gu[3], Dong Wang[1] and Xun Wang[1]*

[1]Key Lab of Organic Optoelectronics and Molecular Engineering, Department of Chemistry, Tsinghua University, Beijing 100084, China. [2]Beijing Synchrotron Radiation Facility, Institute of High Energy Physics, Chinese Academy of Sciences, Beijing 100049, P. R. China. [3]Beijing National Laboratory for Condensed Matter Physics, Institute of Physics, Chinese Academy of Sciences, Beijing 100190, China.
Deren Yang and Hongde Yu contributed equally to this work.

*E-mail: wangxun@mail.tsinghua.edu.cn

Supplementary Methods

Synthesis of TCCP.

All chemicals were purchased commercially and used without additional purification except tetrakis (4-carboxyphenyl)-porphyrin (TCPP) which was synthesized in according to the previous report[1].

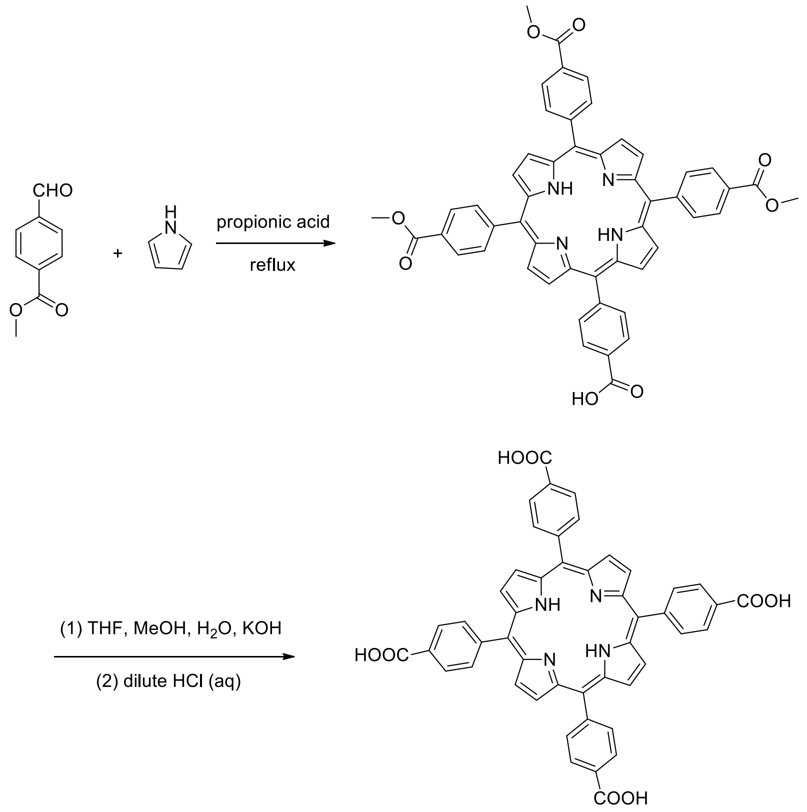

DFT calculations.

The Gibbs free energy change under the zero electrode potential for each reaction step was calculated by the equation $\Delta G=\Delta E+\Delta \mathrm{ZPE}+\Delta_{0\rightarrow298}H-T\Delta S$, in which the metal-coordinated porphyrins were fixed after optimization and thus zero-point vibrational energy (ZPE), thermal energy (H) and entropy (S) contributions of them were excluded from the calculations.

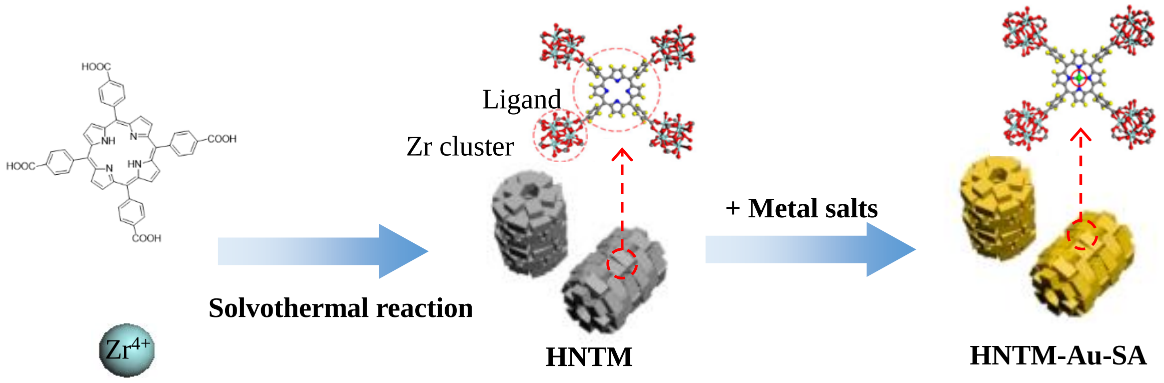

Supplementary Figure 1. Preparation of catalysts. Schematic illustration of preparation of HNTM and HNTM-Au-SA.

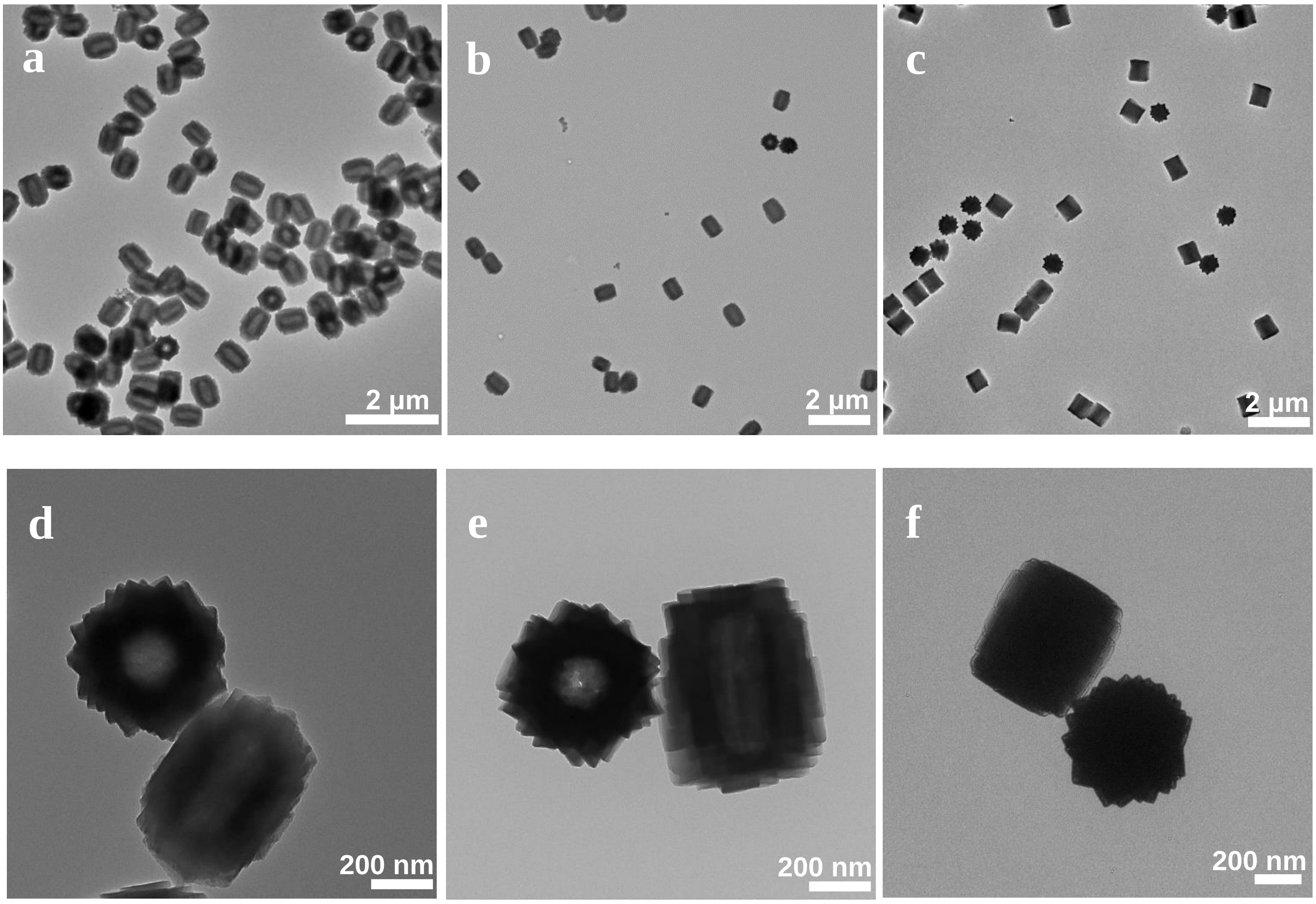

Supplementary Figure 2. Nanostructure characterization of catalysts. TEM image of **a,d,** HNTM, **b,e,** HNTM-Au-SA and **c,f**, HNTM-Au-NP.

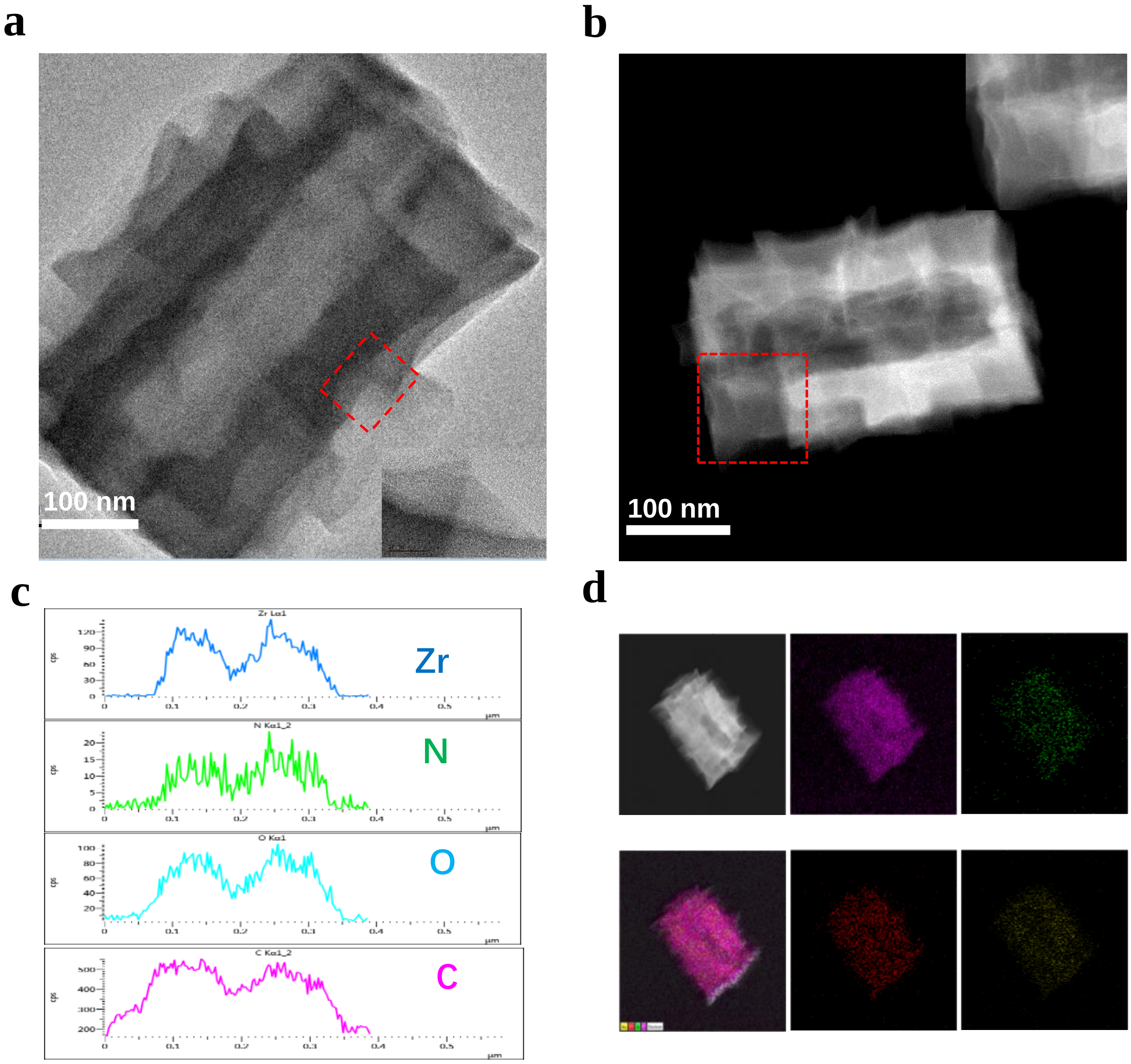

Supplementary Figure 3. Nanostructure characterization of HNTM-Au-SA. a, HRTEM image of HNTM-Au-SA (inset is magnified image of red square). **b,** STEM image of HNTM-Au-SA. (inset is magnified image). **c,** Line-scanning spectra of HRTEM-Au-SA. **d,** EDS elemental mapping of HNTM-Au-SA.

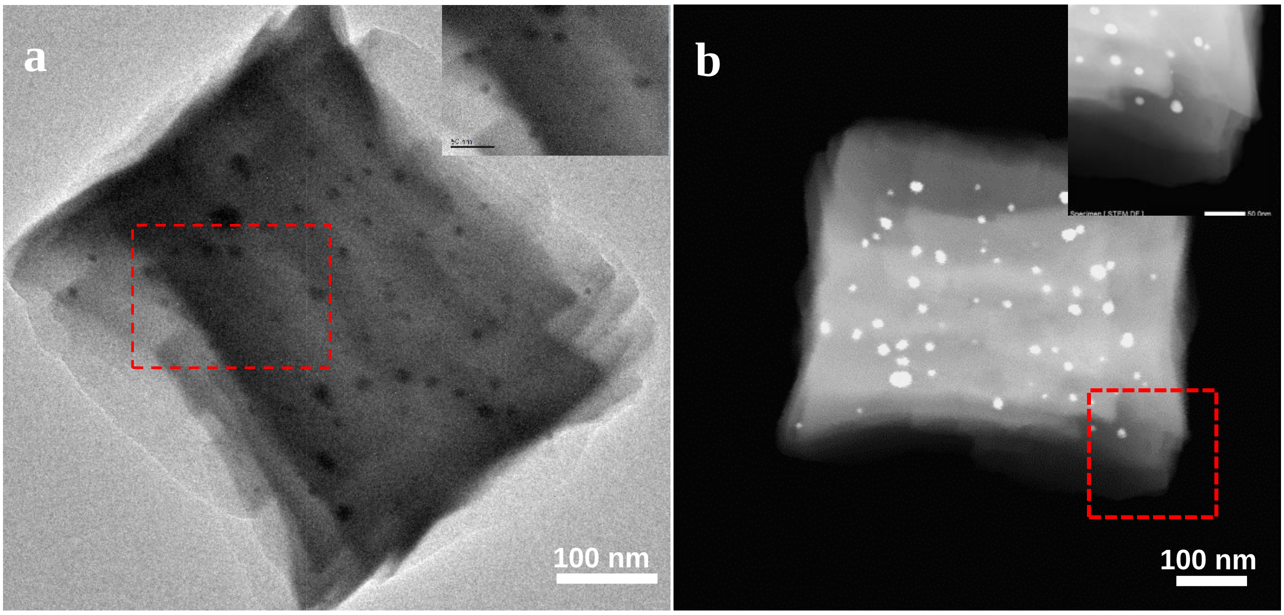

Supplementary Figure 4. Nanostructure characterization of HNTM-Au-NP. a,b, HRTEM image and STEM image of HNTM-Au-NP (inset shows magnified image of red square).

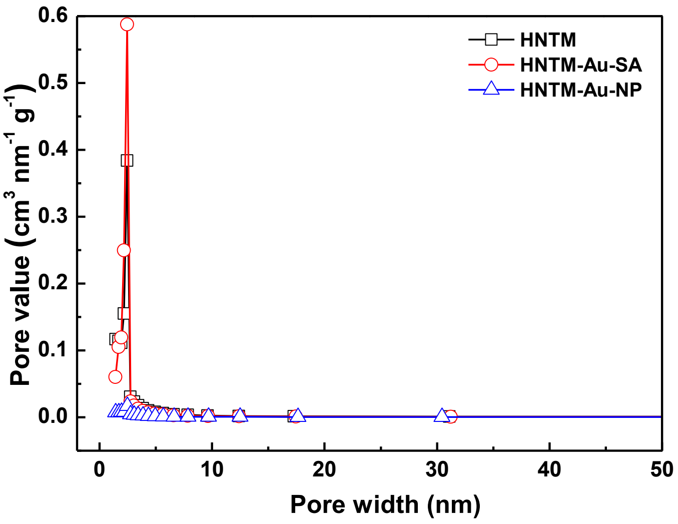

Supplementary Figure 5. Pore size distribution curves of HNTM, HNTM-Au-SA and HNTM-Au-NP.

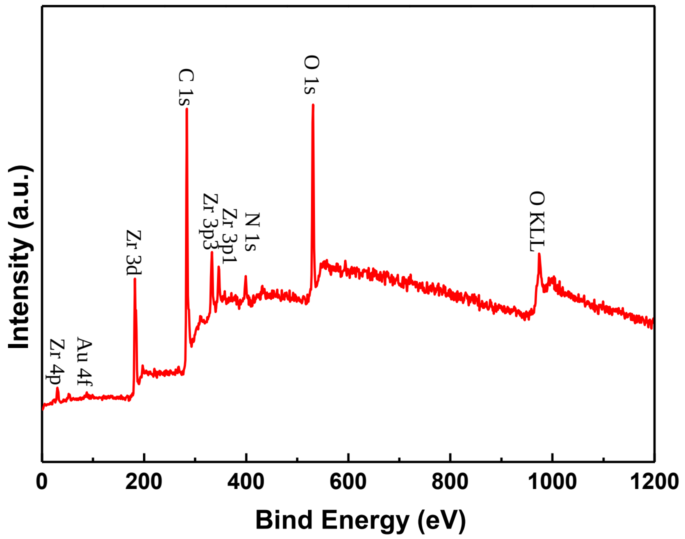

Supplementary Figure 6. XPS spectrum of HNTM-Au-SA.

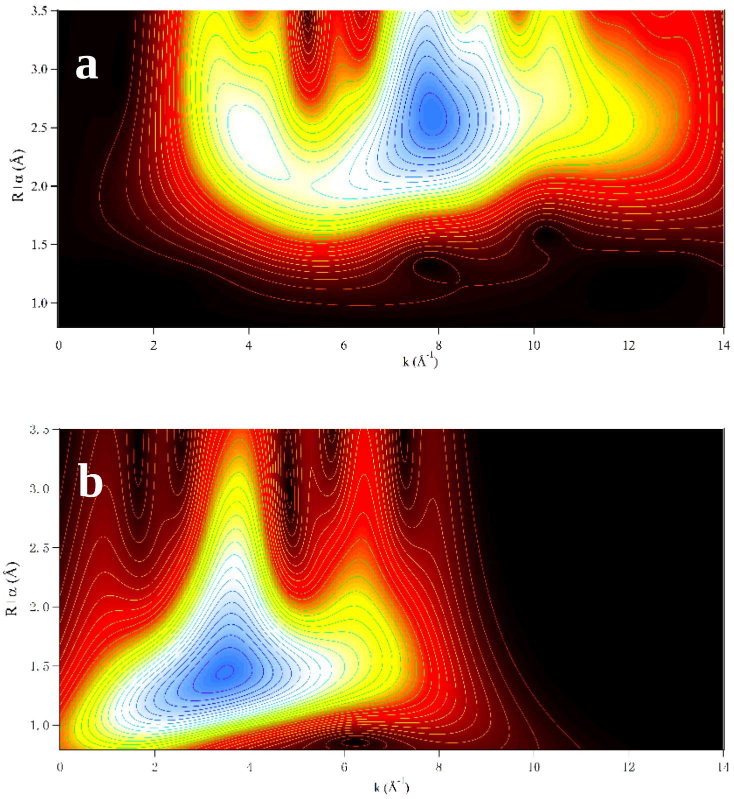

Supplementary Figure 7. Wavelet transform. a,b, Wavelet transform of Au foil and HNTM-Au-SA, respectively.

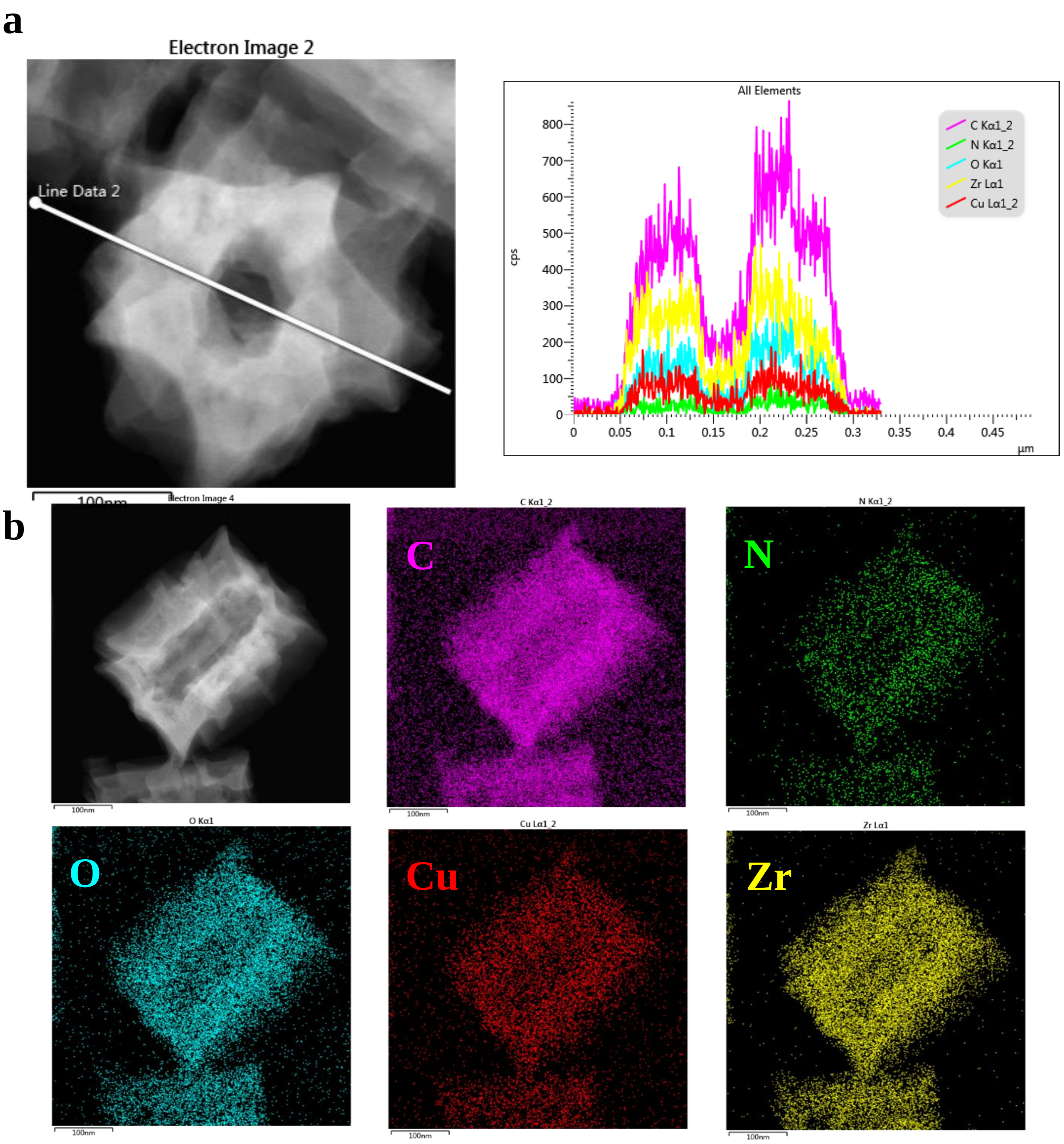

Supplementary Figure 8. Nanostructure characterization of HNTM-Cu-SA. a, Line-scanning spectra of HNTM-Cu-SA. **b,** EDS elemental mapping of HNTM-Cu-SA.

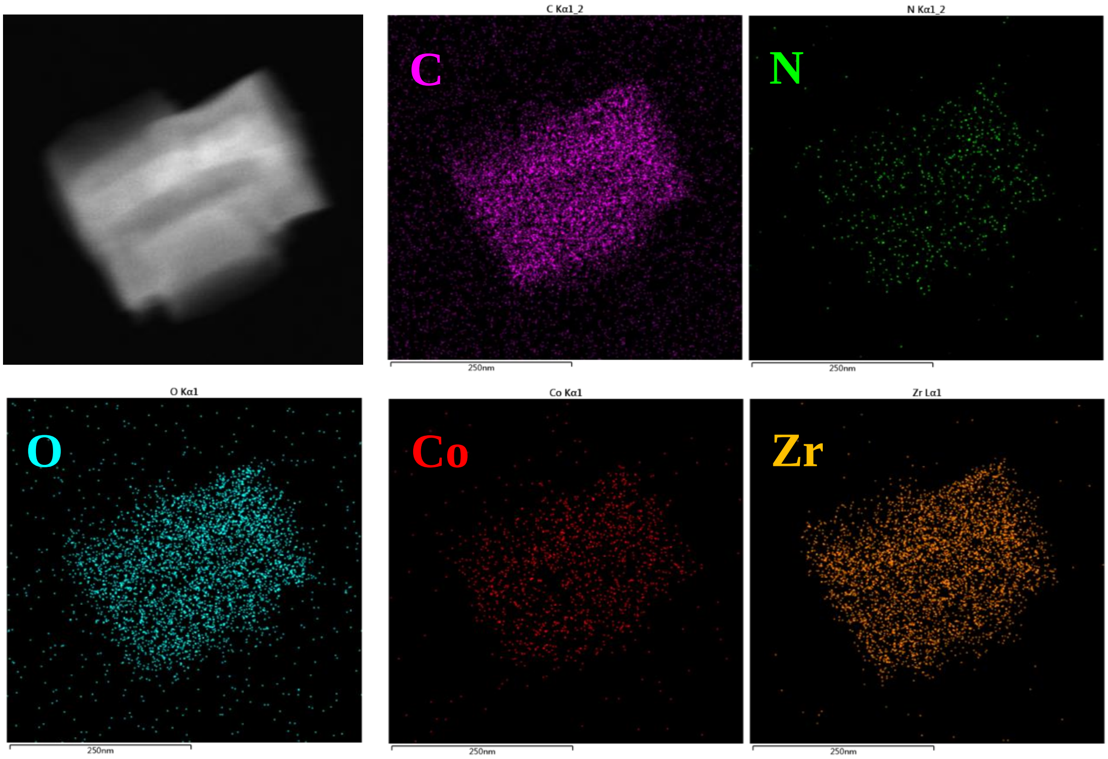

Supplementary Figure 9. EDS elemental mapping of HNTM-Co-SA.

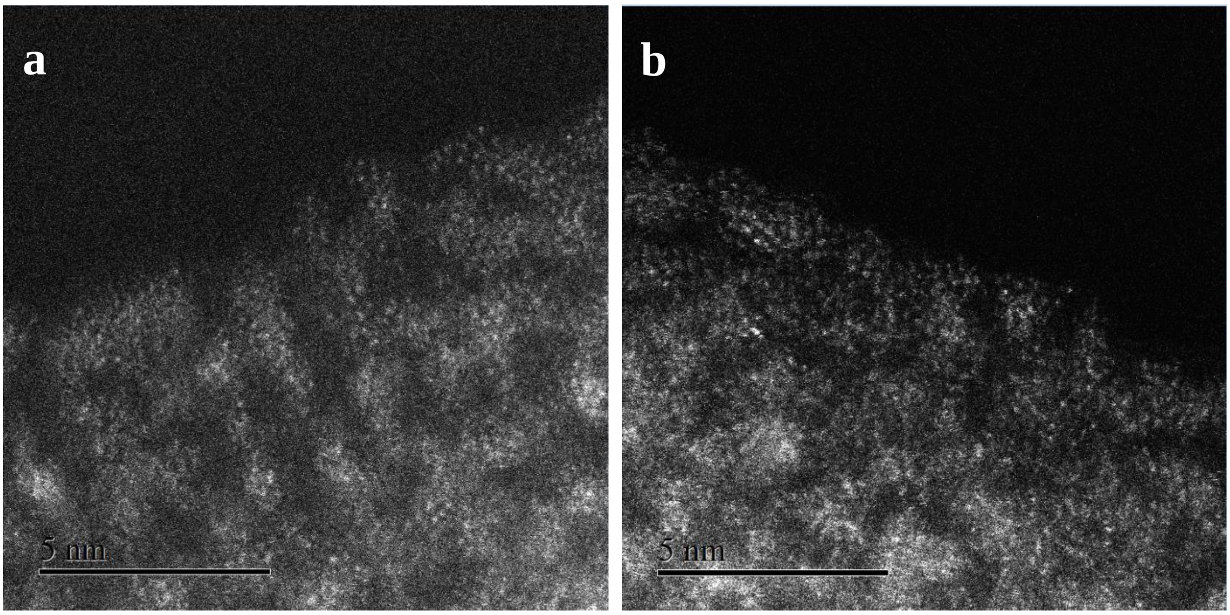

Supplementary Figure 10. Single-atom structure characterization. a,b, HAADF-STEM image of HNTM-Co-SA and HNTM-Cu-SA, respectively.

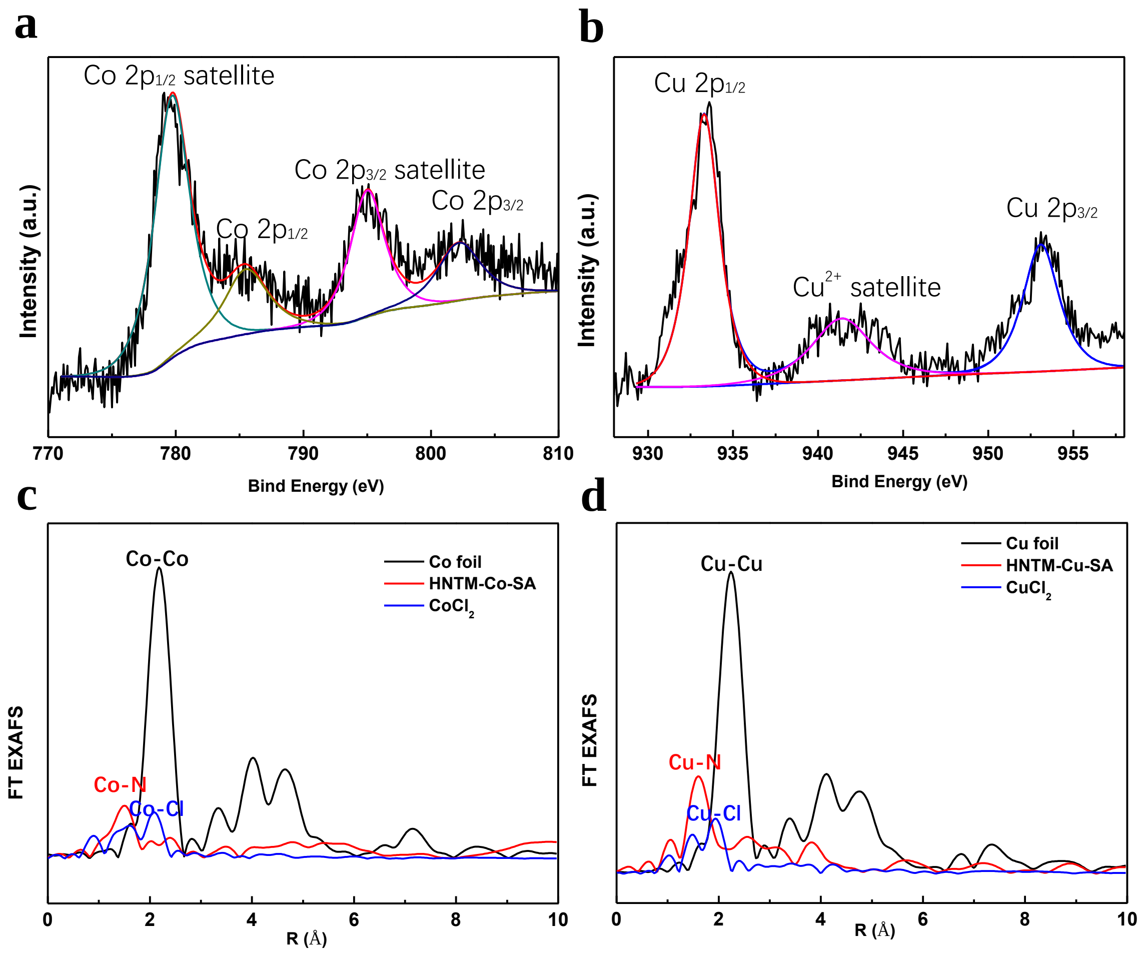

Supplementary Figure 11. Co and Cu atom structure characterization. a, Co 2p XPS spectrum of HNTM-Co-SA. **b,** Cu 2p XPS spectrum of HNTM-Cu-SA. **c,d,** The FT-EXAFS spectra of HNTM-Co-SA and HNTM-Cu-SA at Co and Cu K-edge, respectively.

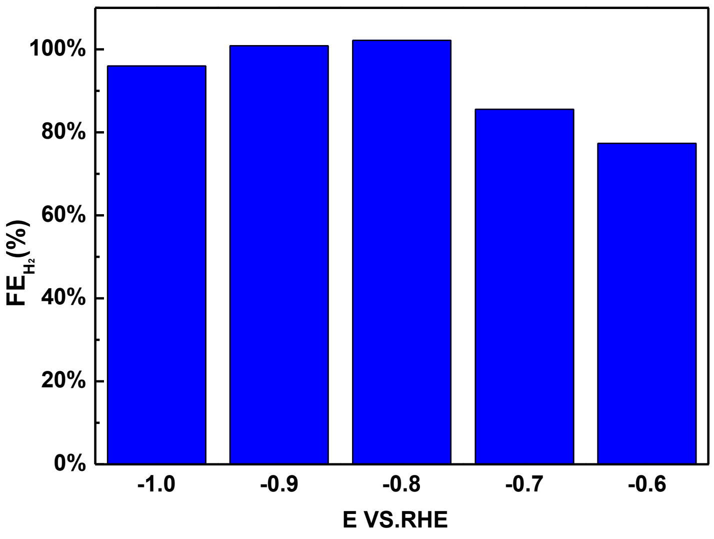

Supplementary Figure 12. FE on HNTM-Au-SA in N_2-saturared 0.1 M $KHCO_3$.

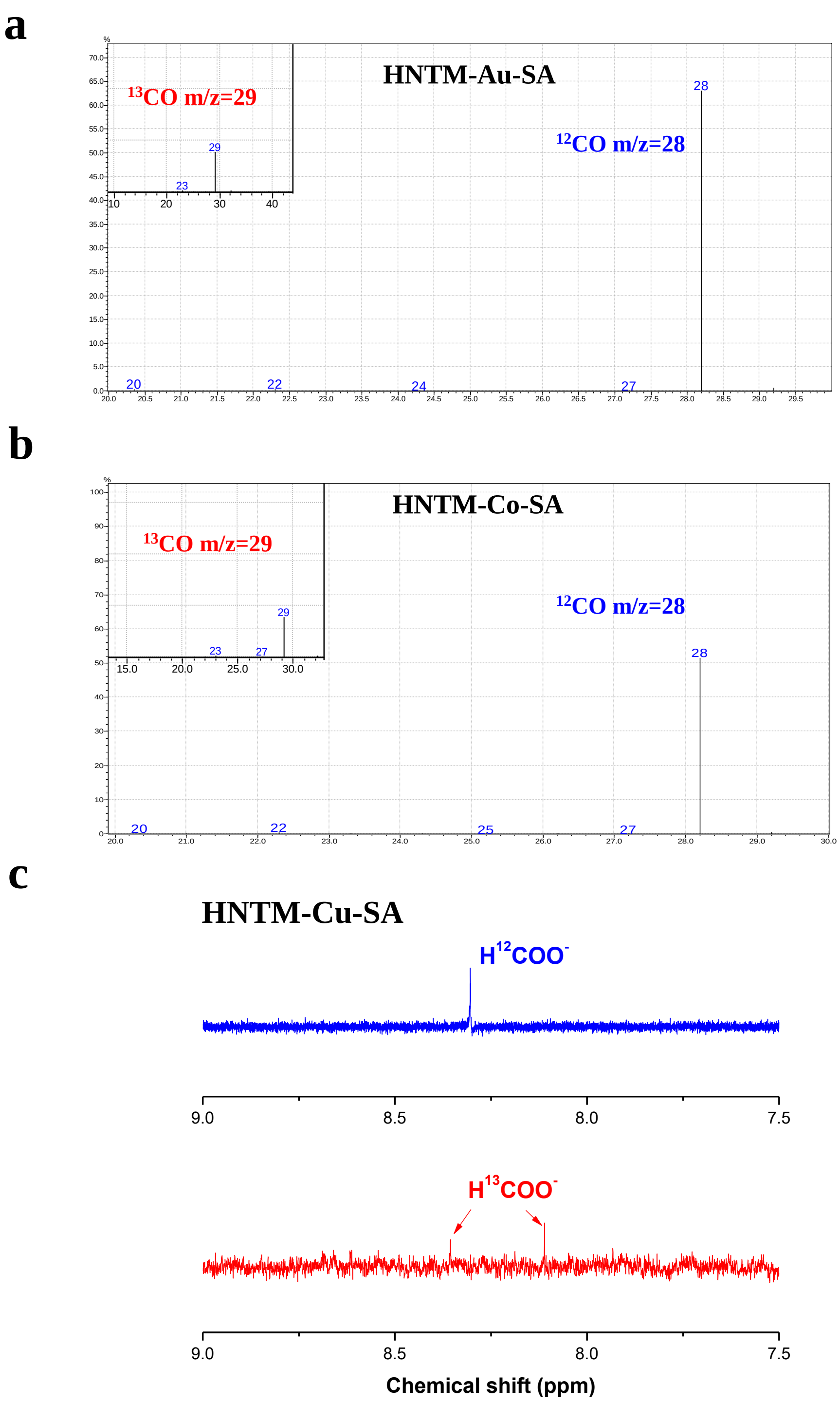

Supplementary Figure 13. Isotope labeling study. a,b, Mass spectra of ^{12}CO (m/z=28) formed on HNTM-Au-SA and HNTM-Co-SA, respectively. Insets show mass spectra of ^{13}CO (m/z=29) when $^{13}CO_2$ is used. **c,** ^1H NMR spectra of the electrolyte after $^{12}CO_2$ (blue spectrum) and $^{13}CO_2$ (red spectrum) electrolysis.

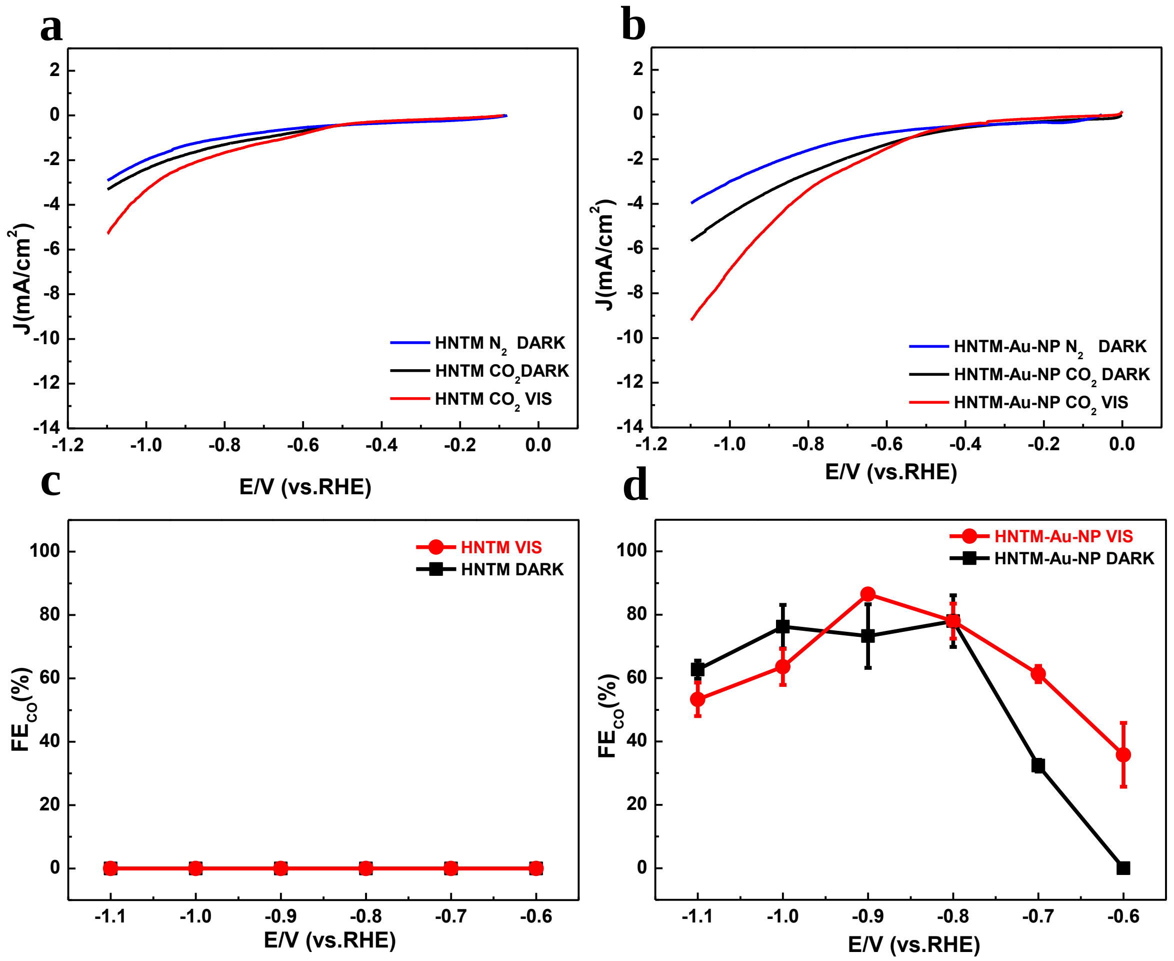

Supplementary Figure 14. Photoelectrochemical performance of CO_2 reduction on HNTM-Co-SA and HNTM-Cu-SA. a,b, LSV curves of HNTM and HNTM-Au-NP respectively in N_2-(blue line)/ CO_2-saturared 0.1 M $KHCO_3$ under visible light (red lines)/dark (black lines). **c,d,** FE_{CO} of HNTM and HNTM-Au-NP respectively under visible light (red lines)/dark (black lines). Error bars are ±s.d.

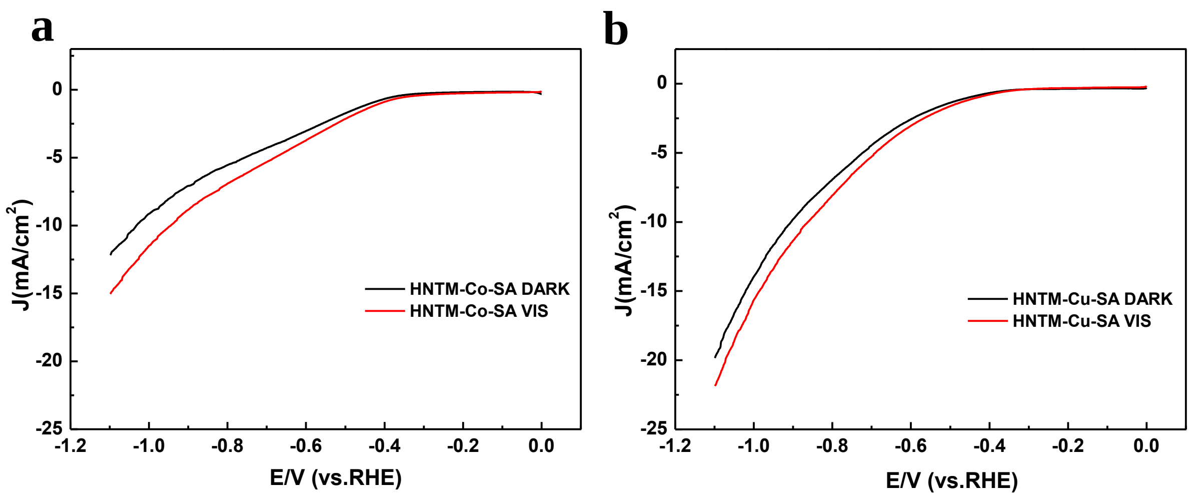

Supplementary Figure 15. LSV measurements of HNTM-Co-SA and HNTM-Cu-SA.

a,b, LSV curves of HNTM-Co-SA and HNTM-Cu-SA respectively under visible light (red lines)/dark (black lines).

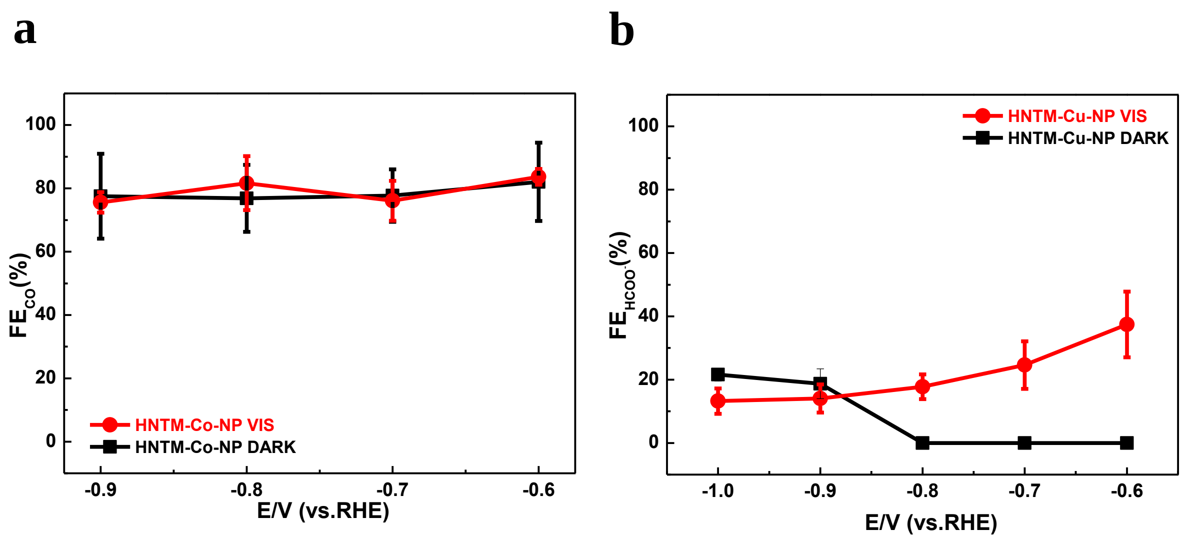

Supplementary Figure 16. FE of HNTM-Co-NP and HNTM-Cu-NP. a,b, FE of HNTM-Co-NP and HNTM-Cu-NP respectively under visible light (red lines)/dark (black lines). Error bars are ±s.d.

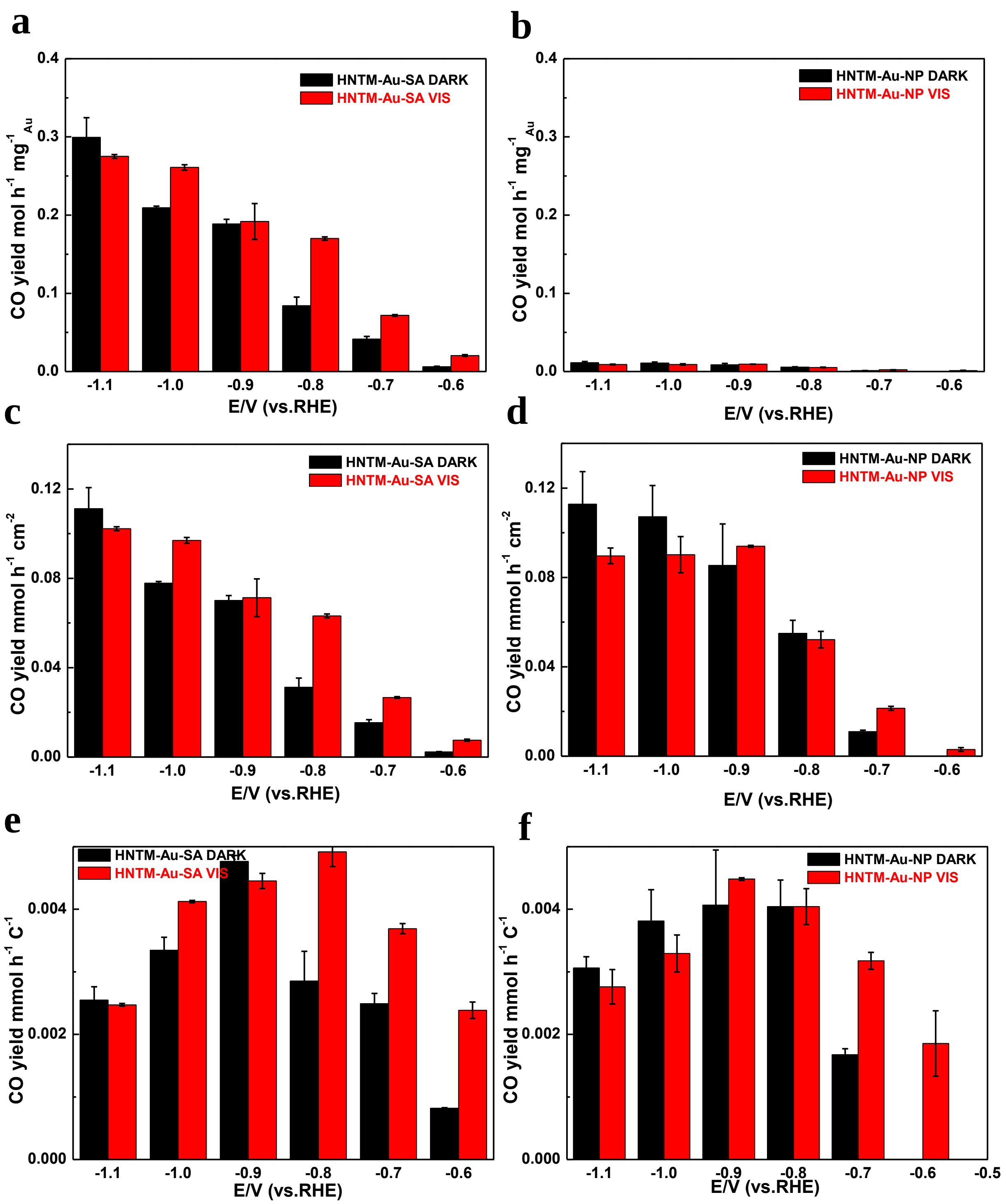

Supplementary Figure 17. CO formation rate on HNTM-Au-SA (left) and HNTM-Au-NP (right). a,c,e, The mass-specific rate, area-specific rate and charge-specific rate of CO on HNTM-Au-SA respectively. **b,d,f,** The mass-specific rate, area-specific rate and charge-specific rate of CO on HNTM-Au-NP respectively under visible light (red bars)/dark (black lines). Error bars are ±s.d.

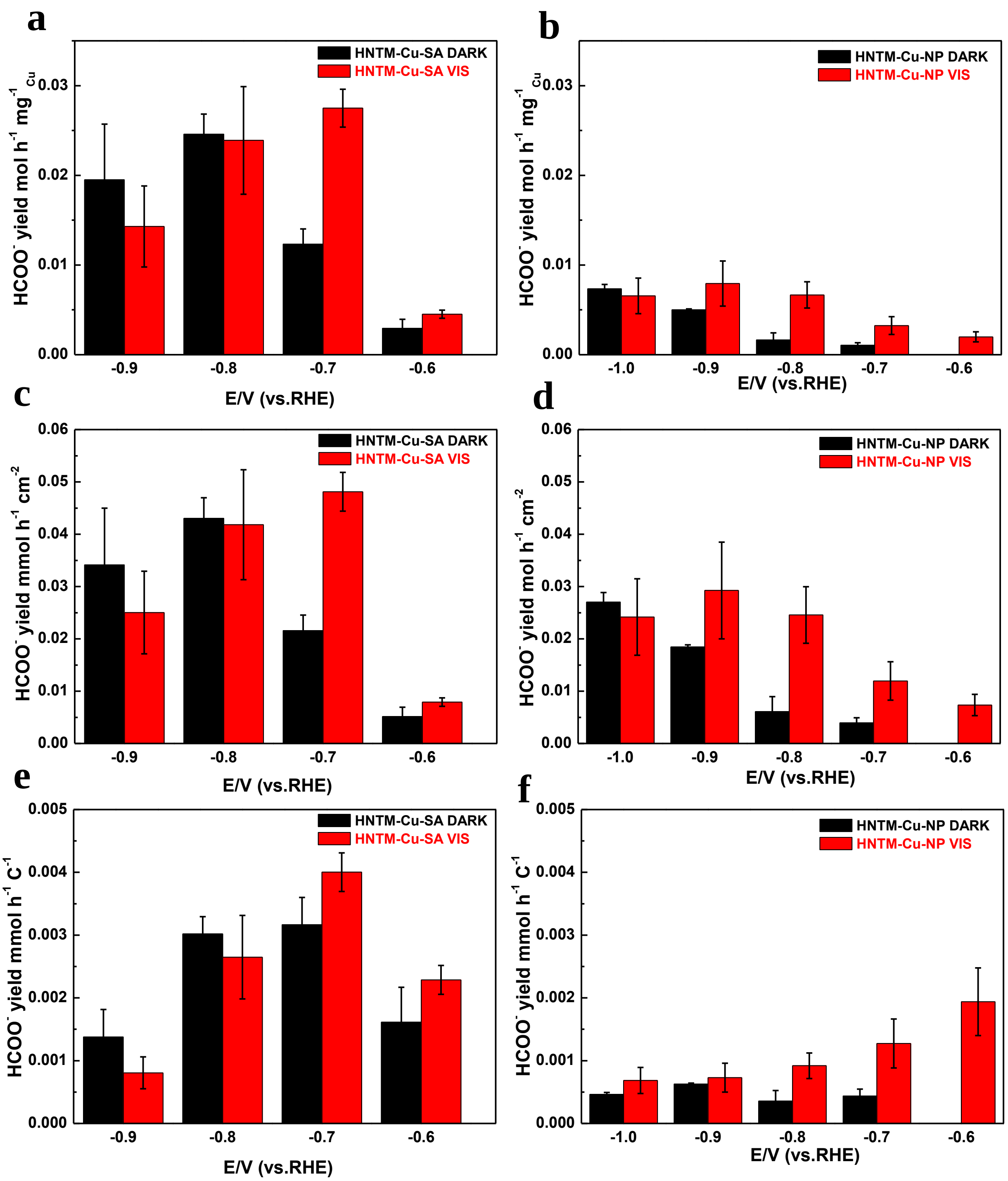

Supplementary Figure 18. $HCOO^-$ formation rate on HNTM-Cu-SA (left) and HNTM-Cu-NP (right). a,c,e, The mass-specific rate, area-specific rate and charge-specific rate of $HCOO^-$ on HNTM-Cu-SA respectively. **b,d,f,** The mass-specific rate, area-specific rate and charge-specific rate of $HCOO^-$ on HNTM-Cu-NP respectively under visible light (red bars)/dark (black lines). Error bars are ±s.d. Error bars are ±s.d.

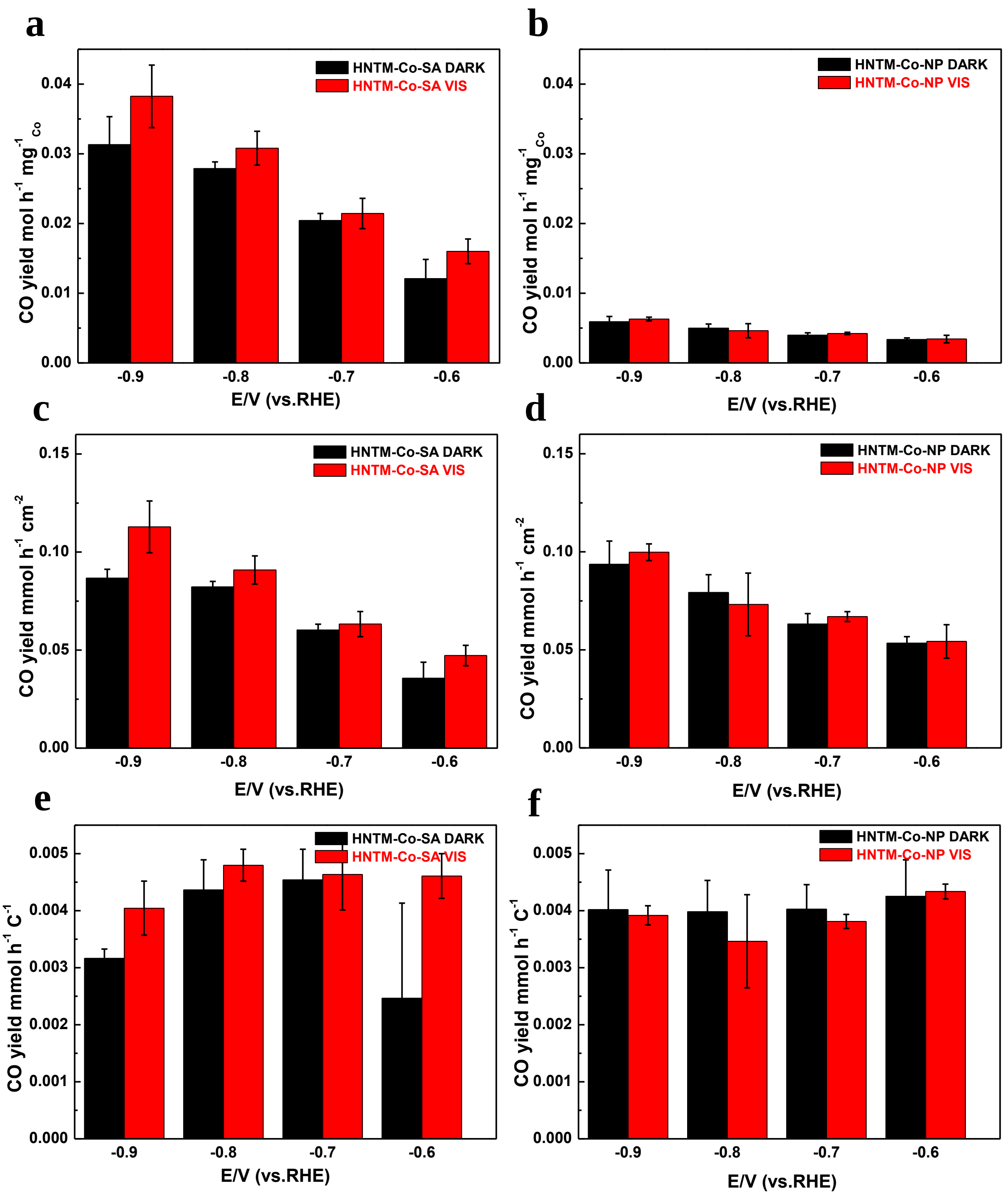

Supplementary Figure 19. CO formation rate on HNTM-Co-SA (left) and HNTM-Co-NP (right). a,c,e, The mass-specific rate, area-specific rate and charge-specific rate of CO on HNTM-Co-SA respectively. **b,d,f,** The mass-specific rate, area-specific rate and charge-specific rate of CO on HNTM-Co-NP respectively under visible light (red bars)/dark (black lines). Error bars are ±s.d. Error bars are ±s.d.

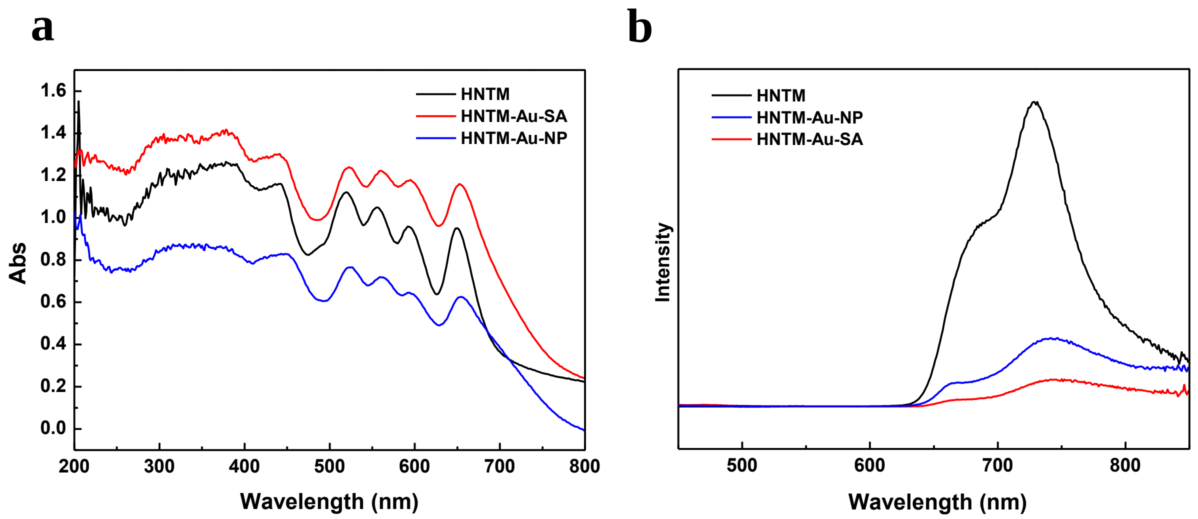

Supplementary Figure 20. Photoelectrochemical measurements of HNTM-Au-SA. a, UV-vis spectra of HNTM, HNTM-Au-SA and HNTM-Au-NP. **b,** PL emission spectra of HNTM, HNTM-Au-NP and HNTM-Au-SA.

Supplementary Table 1. Structural parameters of HNTM-Au-SA and Au foil calculated from the EXAFS fitting.

Samples	Scattering pair	Coordination Number	Bond length	$\sigma^2(10^{-3}\ Å^2)$
HNTM-Au-SA	Au-N	4	1.52	7.85
Au foil	Au-Au	12	2.59	7.28

Error bounds (accuracies) were estimated as Coordination Number, ±5%; Bond length, ±1%;σ^2 (Debye-Waller factor), ±5%.

Supplementary Table 2. Comparision of metal loading on HNMT-M-SA and HNTM-M-NP.

Catalyst	HNTM-Au-SA	HNTM-Au-NP	HNTM-Cu-SA	HNTM-Cu-NP	HNTM-Co-SA	HNTM-Co-NP
Loading amount	0.07%	1.00%	0.35%	2.07%	0.59%	3.17%

Supplementary Table 3. Performance comparision of recently reported electrocatalysts towards CO_2 reduction.

Catalyst		Major Product	electrolyte	Potential (V vs. RHE)	FE	TOF_{max} (h^{-1})	Durability	Ref.
This work	HNTM-Au-SA	CO	0.1 M $KHCO_3$	-0.8	95.2%	37069	90% after 24h	This work
	HNTM-Au-NP	CO	0.1 M $KHCO_3$	-0.8	78.0%	N/A	N/A	This work
	HNTM-Co-SA	CO	0.1 M $KHCO_3$	-0.8	90.4%	1864	N/A	This work
	HNTM-Co-NP	CO	0.1 M $KHCO_3$	-0.8	81.6%	N/A	N/A	This work
	HNTM-Cu-SA	$HCOO^-$	0.1 M $KHCO_3$	-0.7	77.2%	1760	N/A	This work
	HNTM-Cu-NP	$HCOO^-$	0.1 M $KHCO_3$	-0.7	24.6%	N/A	N/A	This work
Porphyrin-based catalyst	Cu-MOF nanosheets	$HCOO^-$	1 M H_2O/CH_3CN solutions with 0.5 M $EMIMBF_4$	-1.55 V vs. Ag/Ag^+	68.4%	2037	5 h	2
	Fe-PB	CO	0.5 M $KHCO_3$	-0.63	100±2%	6280	85% after 24h	3
	CoTPP–CNT	CO	0.5 M $KHCO_3$	-1.35 V vs. SCE	91%	280	a slow catalyst deactivation	4
	CoPP-PG	CO	0.1 M $HClO_4$	-0.6	~60%	2880	N/A	5
Photoelectrocatalyst	$Ru(bpy)_2dppz$-Co_3O_4/CA	$HCOO^-$	0.1 M $NaHCO_3$	-0.6 V vs. NHE	86%	122	8 h	6
	Ag-supported dendritic Cu	hydrocarbons	0.1 M $CsHCO_3$	-1.0 V vs. SCE	79±6%	--	20 days	7
	Cu−Co_3O_4 NTs	$HCOO^-$	0.1 M Na_2SO_4	-0.87	77.5%	--	8 h	8
	FeS_2 /TiO_2 NTs	methanol	0.1 M $KHCO_3$	-1.2 V. SCE	39.8%	--	350 min	9

Supplementary References

1. Feng, D., Gu, Z. Y., Li, J. R., Jiang, H. L., Wei, Z., & Zhou, H. C. Zirconium-metalloporphyrin PCN-222: mesoporous metal-organic frameworks with ultrahigh stability as biomimetic catalysts. *Angew. Chem. Int. Ed.* **51**, 10307-10310 (2012).
2. Wu, J. X. *et al.* Cathodized copper porphyrin metal-organic framework nanosheets for selective formate and acetate production from CO_2 electroreduction. *Chem. Sci.* **10**, 2199-2205 (2019).
3. Smith, P.T. *et al.* Iron Porphyrins Embedded into a Supramolecular Porous Organic Cage for Electrochemical CO_2 Reduction in Water. *Angew. Chem. Int. Ed.* **57**, 9684-9688 (2018).
4. Hu, X. M., Rønne, M. H., Pedersen, S. U., Skrydstrup, T., & Daasbjerg, K. Enhanced Catalytic Activity of Cobalt Porphyrin in CO_2 Electroreduction upon Immobilization on Carbon Materials. *Angew. Chem. Int. Ed.* **56**, 6468-6472 (2017).
5. Shen. J. *et al.* Electrocatalytic reduction of carbon dioxide to carbon monoxide and methane at an immobilized cobalt protoporphyrin. *Nat. Commun.* **6**, 8177 (2015).
6. Huang, X., Shen, Q., Liu, J., Yang, N., & Zhao, G. A CO_2 adsorption-enhanced semiconductor/metal-complex hybrid photoelectrocatalytic interface for efficient formate production. *Energy Environ. Sci.* **9**, 3161-3171 (2016).
7. Gurudayal, G. *et al.* Si photocathode with Ag-supported dendritic Cu catalyst for CO_2 reduction. *Energy Environ. Sci.* **12**, 1068-1077 (2019).
8. Shen, Q., Chen, Z., Huang, X., Liu, M., & Zhao, G. High-Yield and Selective Photoelectrocatalytic Reduction of CO_2 to Formate by Metallic Copper Decorated Co_3O_4 Nanotube Arrays. *Environ. Sci. Technol.* **49**, 5828-5835 (2015).
9. Han, E. *et al.* Worm-like FeS_2/TiO_2 Nanotubes for Photoelectrocatalytic Reduction of CO_2 to Methanol under Visible Light. *Energy Fuels* **32**, 4357-4363 (2018).
